# Case Report: Apparent progressive complete mismatch repair protein loss during platinum-based chemotherapy in metastatic rectal neuroendocrine tumour grade 2

**DOI:** 10.3389/fonc.2026.1885566

**Published:** 2026-09-28

**Authors:** Yue Yang, Gao Li, Jinghui Wang, Wenyu Wu, Dongxin Tang

**Affiliations:** 1 Guizhou University of Traditional Chinese Medicine, Guiyang, Guizhou, China; 2 First Affiliated Hospital of Guizhou University of Traditional Chinese Medicine, Guiyang, Guizhou, China; 3 Affiliated Hospital of Zunyi Medical University, Zunyi, Guizhou, China

**Keywords:** apparent progressive MMR loss, IHC–NGS discordance, Immune checkpoint inhibitor, Microsatellite Instability, Mismatch repair deficiency, Platinum-based chemotherapy, rectal neuroendocrine tumour, surufatinib

## Abstract

**Background:**

Mismatch repair (MMR) deficiency is uncommon in gastroenteropancreatic neuroendocrine tumours (GEP-NETs), yet it represents a critical biomarker for immune checkpoint inhibitor (ICI) eligibility. Treatment-associated reduction or loss of MMR protein expression, often without corresponding MSI-H, has been reported in colorectal adenocarcinoma, but comparable changes have not been described in neuroendocrine tumours. Persistent discordance between immunohistochemistry (IHC)-based MMR protein assessment and next-generation sequencing (NGS)-based microsatellite instability (MSI) testing is an emerging and clinically challenging phenomenon.

**Case presentation:**

We report a 55-year-old woman initially diagnosed with rectal NET Grade 1 on endoscopic biopsy; the tumour was upgraded to NET Grade 2 (Ki-67 approximately 20%) on the surgical resectionk specimen. Hepatic metastases were confirmed four months after resection. The first liver biopsy (January 16, 2026) demonstrated partial MMR protein loss by IHC (MLH1-, MSH2+, MSH6-, PMS2-), while concurrent NGS showed microsatellite stability (MSS) and a tumour mutational burden (TMB) of <1 mut/Mb. The patient received two cycles of surufatinib 250 mg orally once daily combined with intravenous carboplatin (500 mg, day 1) and etoposide (160 mg, days 1-3). Restaging CT demonstrated disease progression. A repeat liver biopsy (March 17, 2026) revealed complete loss of all four MMR proteins by IHC (MLH1-, MSH2-, MSH6-, PMS2-), while NGS again confirmed MSS and TMB <1 mut/Mb.

**Conclusions:**

To the best of our knowledge, this is the first reported case of apparent progressive complete MMR protein loss in the context of sequential platinum-based chemotherapy in metastatic rectal NET Grade 2, with persistent IHC-NGS discordance throughout. These findings raise an important question regarding how IHC-defined dMMR should be interpreted in the absence of NGS-confirmed MSI-H, and highlight the complexity of immunotherapy eligibility decisions in rare tumour types. Prospective data are needed to guide clinical practice.

## 1 Introduction

Rectal neuroendocrine tumours (NETs) are rare but increasingly diagnosed, with a reported detection rate of 0.17% in screening colonoscopy cohorts (1, 2). Analysis of US SEER data showed that the overall incidence of NENs increased 5.2-fold between 1975 and 2021, whereas the incidence of rectal NENs increased 12-fold; this overall rise was driven largely by increased diagnosis of early-stage disease (3). Small, well-differentiated rectal NETs confined to the submucosa, particularly those ≤10 mm, usually have an excellent prognosis after complete endoscopic resection (4). Histological grade is defined by proliferative activity, including the Ki-67 index and mitotic count (5), whereas increasing tumour size and poorer differentiation are associated with a higher metastatic risk (6). Once hepatic or other distant metastases occur, prognosis worsens substantially; contemporary SEER data indicate a 10-year overall survival of 15.4% for distant-stage, well-differentiated NETs of rectal origin (3).

The systemic treatment landscape for metastatic rectal NETs is evolving and remains largely extrapolated from broader GEP-NET evidence. For most patients with metastatic G1-G2 well-differentiated gastrointestinal NETs, SSAs are recommended as first-line systemic therapy, whereas everolimus is an established subsequent-line option, particularly for non-functioning or SSTR-negative tumours (7). Surufatinib is a novel oral agent that simultaneously targets vascular endothelial growth factor receptors (VEGFR-1/2/3), fibroblast growth factor receptor 1 (FGFR-1), and the macrophage colony-stimulating factor 1 receptor (CSF-1R); in the phase 3 SANET-ep and SANET-p trials, it significantly improved progression-free survival compared with placebo in patients with extrapancreatic and pancreatic NETs (8, 9). Platinum-etoposide regimens are commonly used for poorly differentiated NECs (10). Although cytotoxic chemotherapy may be considered for selected patients with rapidly progressive or high-volume well-differentiated G2/G3 NETs, evidence supporting platinum-etoposide specifically in this setting, particularly for rectal primaries, remains limited (7).

MMR deficiency and the resultant microsatellite instability-high (MSI-H) phenotype have become pan-tumour predictive biomarkers for ICI response. In 2017, the US FDA granted accelerated approval to pembrolizumab for MSI-H/dMMR solid tumours, followed subsequently by approval from the National Medical Products Administration in China. It should be emphasised that, in GEP-NENs, the frequency of MSI-H/dMMR and TMB-H is closely related to histological differentiation and primary site: these phenotypes are rare in well-differentiated or low-grade GEP-NETs, but relatively enriched in high-grade or poorly differentiated gastrointestinal NEC/MiNEN. Large molecular profiling cohorts have shown MSI-H and TMB-H frequencies of approximately 0% and 1%, respectively, in low-grade GEP-NENs, compared with approximately 4% and 7% in high-grade tumours (11); another gastrointestinal NEC/MANEC cohort reported MSI-H in approximately 12.4% of cases (12). In a prospective, non-MSI-H/TMB-H-enriched cohort of well-differentiated NETs, single-agent pembrolizumab showed limited activity, with an objective response rate of only 3.7% in the KEYNOTE-158 NET cohort (13). Therefore, guideline recommendations such as those from NCCN supporting pembrolizumab for metastatic NET/NEN with MSI-H/dMMR or TMB-H are largely extrapolated from pan-tumour ICI data in the absence of NET-specific prospective trials.

A clinically important but under-recognised challenge is discordance between IHC-based MMR protein assessment and NGS-based MSI testing. A large concordance study of NGS-MSI and IHC-MMR across more than 190,000 solid tumour samples found that approximately 0.6% of cases were initially flagged as discordant; after pathologist re-evaluation, the final discordance rate was 0.31% (14). A separate French multicentre study of 3,228 colorectal cancer patients reported an initial discordance rate of 1.6%, related to both IHC and molecular testing misclassification (15). The biological and therapeutic implications of persistent IHC-dMMR/NGS-MSS remain poorly understood, particularly in rare histological types such as NETs. Platinum-resistance-associated hypermethylation of the hMSH2 upstream regulatory region has been reported in ovarian cancer (16), whereas treatment-associated reduction or loss of MMR protein expression with persistent MSS has been reported in colorectal cancer after preoperative therapy (17). To our knowledge, comparable treatment-associated MMR changes have not been reported in NETs.

Herein, we report a case of metastatic rectal NET Grade 2 in which sequential liver biopsies obtained before and after two cycles of platinum-containing systemic therapy showed an apparent change from partial MMR protein loss to loss of all four MMR proteins by IHC, while NGS consistently confirmed MSS. This case illustrates temporal variation in MMR protein expression during systemic therapy and raises clinically relevant diagnostic and therapeutic questions for the management of advanced NETs.

## 2 Case presentation

### 2.1 Initial presentation and diagnosis

A 55-year-old Han Chinese woman with a 20-year history of essential hypertension (managed with amlodipine besylate 5 mg daily; baseline blood pressure approximately 165/85 mmHg, subsequently well controlled) presented on September 5, 2025 with a four-day history of haematochezia. She denied smoking, alcohol use, or a family history of malignancy. Colonoscopy at a referring institution identified a rectal mass with concurrent internal haemorrhoids. Endoscopic biopsy showed sparse intestinal mucosal epithelium and glandular elements, with small cells showing scant cytoplasm in the stroma. IHC (report C202513384) supported a diagnosis of rectal NET Grade 1: CK20(-), CK7(-), CK5/6(-), CD56(+), Ki-67 2%, P40(-), PCK(+), SYN(+), P63(-), CgA(-); mitotic index 1/2 mm^2^.

Rectal MRI demonstrated a mid-rectal mass staged mrT3bN2M0 with positive mesorectal fascia involvement (MRF+) and extramural vascular invasion (EMVI+). Staging CT of the chest, abdomen, and pelvis revealed bilateral small pulmonary nodules, right renal cysts, and multiple pelvic lymph nodes, without definite evidence of distant metastasis at that time. The patient was subsequently referred to our institution for further management.

### 2.2 Surgical resection and pathological upgrade

On September 17, 2025, the patient underwent laparoscopic-assisted anterior resection of the rectum with diverting ileostomy and adhesiolysis under general anaesthesia. The final surgical pathology report (C202513774) revealed a critical pathological upgrade to NET Grade 2: the primary tumour measured 25 mm in greatest dimension, with approximately 6 mitoses per 2 mm^2^ (C202513774-4), a Ki-67 index of approximately 20%, and invasion into the subserosa. Both anastomotic margins were tumour-free. Of 12 perirectal lymph nodes examined, 1 showed metastatic involvement (1/12); 4 tumour deposits were identified in perirectal adipose tissue. All 3 mesenteric root lymph nodes were negative (0/3). S-100 staining did not indicate perineural invasion (C202513774-7).

MMR protein IHC was not performed on this primary resection specimen. Retrospective staining was considered; however, the archived FFPE tissue blocks had been exhausted by serial sectioning during the original diagnostic workflow, and no residual material remained for additional IHC. Consequently, baseline MMR status could not be assessed retrospectively; this limitation is addressed further in Sections 3.3.2 and 3.4.

The patient recovered uneventfully after surgery and was discharged with a post-operative stage of pT3N1aM0 (AJCC/ENETS).

### 2.3 Detection of hepatic metastases and first liver biopsy

In January 2026, the patient was re-admitted for consideration of ileostomy reversal. Abdominal CT demonstrated diffusely reduced hepatic parenchymal density consistent with hepatic steatosis; multiple new nodular lesions appeared in the right hepatic lobe and showed gradual homogeneous enhancement after contrast administration, a pattern compatible with hypervascular NET metastases (**Figure 1B**). The index lesion in segment S8 measured approximately 22.74 x 23.33 mm. Mild enlargement of left pelvic lymph nodes was also noted, raising the possibility of extrahepatic nodal involvement. Enhanced upper abdominal CT confirmed multiple new right hepatic lobe lesions highly suspicious for metastatic disease.

**Figure 1:**
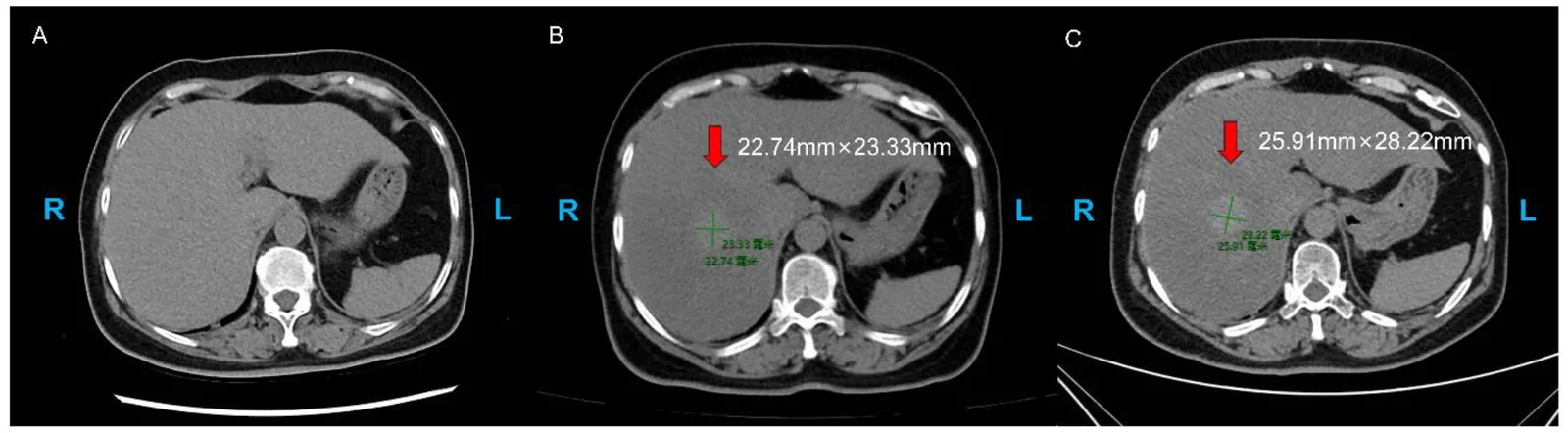
Serial hepatic CT imaging demonstrating disease progression. **(A)** Plain CT of the upper abdomen (October 2025), obtained approximately six weeks after surgical resection, showing a homogeneous hepatic parenchyma without identifiable focal lesions; no evidence of distant metastasis at this time point. **(B)** Contrast-enhanced CT (January 2026) demonstrating a newly identified hypodense nodular lesion in the right hepatic lobe (red arrow; approximately 22.74 mm x 23.33 mm), consistent with a hypervascular neuroendocrine tumour metastasis. **(C)** Restaging contrast-enhanced CT (March 2026), obtained after two cycles of surufatinib plus carboplatin and etoposide, shows interval enlargement of the index lesion (red arrow; approximately 25.91 mm x 28.22 mm).

Ultrasound-guided percutaneous liver biopsy was performed on January 16, 2026. Histopathology (C202600760) confirmed malignant tumour within the hepatic parenchyma. A supplementary IHC report (January 22, 2026) established the diagnosis of metastatic NET Grade 2 (Ki-67 <20%, approximately 10%): CK7(−), CK20(−), CK(+), CgA (weakly+), CD56(+), Ki-67 approximately 10%, SYN(+), CK19(+), Hepatocyte(−), GPC-3(−), AFP(−), HER-2(0). Critically, MMR protein IHC demonstrated partial loss: MLH1(−), MSH2(+), MSH6(−), PMS2(−) (**Figure 2A**).

**Figure 2:**
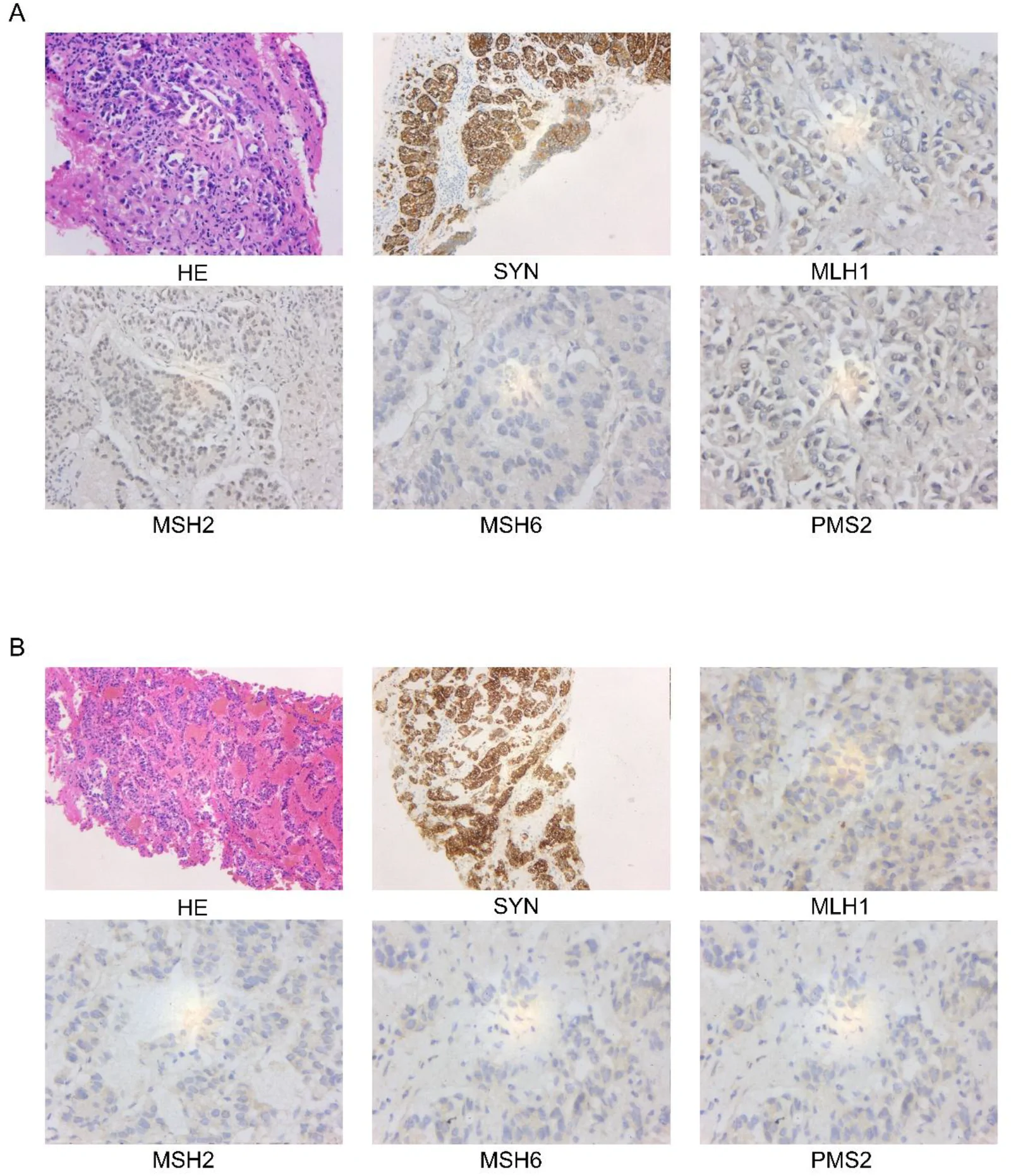
Histopathology and immunohistochemistry of serial liver biopsies demonstrating apparent progressive MMR protein loss. **(A)** First liver biopsy (C202600760, January 2026), prior to systemic chemotherapy. Haematoxylin and eosin (H&E) staining shows tumour cells arranged in nested and trabecular patterns with uniform round nuclei and granular chromatin, consistent with a well-differentiated neuroendocrine tumour. Synaptophysin (SYN) immunostaining demonstrates diffuse strong cytoplasmic positivity, confirming neuroendocrine differentiation. MMR protein immunostaining reveals partial loss: MLH1 expression is absent in tumour cell nuclei; MSH2 expression is retained in tumour cell nuclei (positive); MSH6 and PMS2 expression are absent in tumour cell nuclei. **(B)** Second liver biopsy (C202603763, March 2026), following two cycles of surufatinib plus carboplatin and etoposide. H&E staining demonstrates persistent well-differentiated neuroendocrine tumour morphology. SYN immunostaining confirms retained neuroendocrine differentiation. MMR protein immunostaining now demonstrates complete loss of all four proteins: MLH1, MSH2, MSH6, and PMS2 expression are all absent in tumour cell nuclei.

Concurrent NGS was performed using a validated 500+ gene panel (Genecast BaiShiBo Plus; report P388361-1, January 29, 2026) on paired tumour tissue (collected January 16, 2026) and matched peripheral blood leucocytes (January 23, 2026). The analysis demonstrated: MSS by NGS-based MSI algorithm; TMB <1 mut/Mb (9th percentile); PD-L1 TPS <1%, CPS <1. No clinically actionable somatic variants were identified. No somatic mutations were detected in MMR pathway genes (MLH1, MSH2, MSH6, PMS2). A germline heterozygous variant in TSHR (p.R450H) was incidentally identified, unrelated to MMR. Plasma circulating tumour DNA (ctDNA) NGS (report P388361-2, February 2, 2026; ctDNA fraction <2%) similarly showed MSS and TMB <1 mut/Mb with no somatic variants detected.

The discordance between IHC-defined partial MMR protein loss (MLH1−, MSH2+, MSH6−, PMS2−) and NGS-confirmed MSS was documented and referred for multidisciplinary discussion. The final oncological staging at this point was pT3N1aM1 (Stage IV), consistent with post-operative rectal NET with regional lymph-node and hepatic metastases.

### 2.4 First-line systemic treatment

Following exclusion of contraindications, the patient received two cycles of surufatinib 250 mg orally once daily (continuous dosing) combined with intravenous etoposide 160 mg on days 1–3 and intravenous carboplatin 500 mg on day 1 of each 21-day cycle, between January 23 and February 22, 2026. Treatment was completed without dose reduction. The patient reported mild scalp pruritus, scattered skin rash, fatigue, and occasional palpitations, but no fever, nausea, or vomiting. No haematological toxicity requiring transfusion or growth factor support was documented.

The rationale for this combination regimen reflected the rapidly progressive clinical course, the absence of SSTR imaging data precluding PRRT assessment, and multidisciplinary consensus to extrapolate platinum-etoposide evidence from poorly differentiated or anaplastic NECs (10, 18), despite the limited direct evidence in well-differentiated G2 NETs. Surufatinib was used at 250 mg rather than the 300 mg dose employed in the SANET trials, based on individual tolerance and clinician judgement. This combination regimen is not a current standard of care, and its use represents an extrapolation beyond established guideline recommendations, undertaken given the clinical urgency.

### 2.5 Restaging and second liver biopsy

Restaging upper abdominal CT in March 2026 demonstrated worsening hepatic parenchymal heterogeneity with further reduction in parenchymal density. The index hepatic lesion had enlarged to 25.91 × 28.22 mm (from 22.74 × 23.33 mm at January 2026), with reduced lesion-to-background contrast attributable to both tumour growth and progressive hepatic steatosis (**Figure 1C**). These findings were interpreted radiologically as disease progression.

The patient was admitted on March 16, 2026 for a third treatment cycle. A repeat percutaneous liver biopsy was performed on March 17, 2026. Histopathology (C202603763, reported March 19, 2026) confirmed metastatic NET Grade 2. Strikingly, repeat MMR IHC now demonstrated complete loss of all four MMR proteins: MLH1(−), MSH2(−), MSH6(−), PMS2(−); HER-2(0) (**Figure 2B**).

Repeat NGS on the new hepatic biopsy specimen (P388361-3, March 25, 2026) with matched germline DNA again demonstrated: MSS; TMB <1 mut/Mb (9th percentile); no actionable somatic variants; no somatic mutations in MLH1, MSH2, MSH6, or PMS2; and the same germline TSHR p.R450H variant. The apparent change from partial MMR protein loss (January 2026, prior to chemotherapy) to complete four-protein loss (March 2026, following two cycles of carboplatin and etoposide plus surufatinib), against a background of persistent NGS-confirmed MSS, constitutes the central observation of this report.

At the most recent follow-up (March 29, 2026), the patient maintained a KPS of 80 with an NRS pain score of 0 and no weight loss over the preceding month. The ileostomy was functioning normally. The question of ICI eligibility in the setting of IHC-dMMR/NGS-MSS was under active multidisciplinary tumour board consideration. The longitudinal pathological and molecular findings and the overall clinical timeline are summarised in **Tables 1** and **2**, respectively.

**Table 1:** Longitudinal molecular profiling: summary of pathological grading, MMR IHC status, and NGS findings across biopsy time points.

Time point	Sample source	Ki-67/mitotic index	Grade	MMR IHC (MLH1/MSH2/MSH6/PMS2)	NGS/additional findings
Sep 2025 (Endoscopic biopsy)	Rectal primary (pre-op biopsy)	Ki-67 2% Mitoses: 1/2 mm²	NET G1	Not performed	MRI: mrT3bN2M0, MRF(+), EMVI(+) CT: no definite distant metastasis
Sep 2025 (Surgical specimen)	Rectal primary (resection)	Ki-67 ~20% Mitoses: ~6/2 mm²	NET G2 (upgraded)	Not performed	pT3N1aM0; 1/12 LN positive; 4 tumour deposits; no perineural invasion (S-100−)
Jan 2026 (1st liver biopsy)	Hepatic metastasis	Ki-67 ~10% (<20%)	NET G2 (metastatic)	MLH1 (−) MSH2 (+) MSH6 (−) PMS2 (−) [Partial loss]	NGS (500+ gene panel, tumour + WBC): MSS; TMB <1 mut/Mb (9th percentile); PD-L1 TPS <1%, CPS <1; No actionable somatic variants; Germline TSHR p.R450H (het). Plasma ctDNA: MSS, TMB <1 mut/Mb, no somatic variants detected
Mar 2026 (2nd liver biopsy)	Hepatic metastasis (post 2 cycles carboplatin and etoposide + surufatinib)	Not re-assessed	NET G2 (metastatic)	MLH1 (−) MSH2 (−) MSH6 (−) PMS2 (−) [Complete loss]	NGS (paired tumour + WBC): MSS; TMB <1 mut/Mb (9th percentile); No MMR gene somatic mutations detected; No new actionable variants; Same germline TSHR p.R450H

NET, neuroendocrine tumour; IHC, immunohistochemistry; MMR, mismatch repair; NGS, next-generation sequencing; MSS, microsatellite stable; TMB, tumour mutational burden; LN, lymph node; WBC, white blood cell (germline); ctDNA, circulating tumour DNA; het, heterozygous. (+), protein expression retained; (−), protein expression lost.

**Table 2:** Clinical timeline of key events.

Date	Event	Key findings/actions
Sep 5, 2025	Symptom onset & first endoscopy	Rectal mass identified; biopsy → NET G1 (Ki-67 2%)
Sep 15, 2025	Pre-operative workup at our centre	Pathology confirmed NET G1; MRI: mrT3bN2M0
Sep 17, 2025	Laparoscopic-assisted anterior resection + ileostomy	Surgical specimen upgraded to NET G2 (Ki-67 ~20%); pT3N1a; 1/12 LN positive
Jan 12, 2026	Re-admission (stoma reversal consideration)	CT: new hepatic nodules in right lobe → suspected metastases
Jan 14, 2026	Enhanced CT upper abdomen	Multiple right hepatic lobe nodules; highly suspicious for NET metastases
Jan 16–29, 2026	1st liver biopsy + IHC + NGS	Confirmed metastatic NET G2 (Ki-67 ~10%); IHC: partial MMR loss (MLH1−, MSH2+, MSH6−, PMS2−); NGS: MSS, TMB <1 mut/Mb
Jan 23, 2026	Cycle 1: Surufatinib + Carboplatin/Etoposide	Surufatinib 250 mg po qd + Etoposide 160 mg d1–3 iv + Carboplatin 500 mg d1 iv
Feb 22, 2026	Cycle 2: Surufatinib + Carboplatin/Etoposide	Same regimen; tolerated without dose reduction
Mar 16, 2026	Re-staging CT	CT: disease progression over a 60-day interval (index lesion increased by approximately 5 mm; reduced lesion-to-background contrast)
Mar 17–25, 2026	2nd liver biopsy IHC + NGS	IHC: complete MMR protein loss (MLH1−, MSH2−, MSH6−, PMS2−); NGS: MSS, TMB <1 mut/Mb; no MMR gene somatic mutations

NET, neuroendocrine tumour; LN, lymph node; CT, computed tomography; IHC, immunohistochemistry; NGS, next-generation sequencing; MSS, microsatellite stable; TMB, tumour mutational burden.

## 3 Discussion

### 3.1 Rectal NETs: biological behaviour and prognostic considerations

Low-grade rectal neuroendocrine tumours (G1/G2) mostly arise from hindgut L cells that secrete PYY/GLP-1, and their immunophenotype is characterised by diffuse SYN and CD56 positivity with frequent CgA negativity, consistent with the immunophenotype in the present case. Compared with typical midgut NETs, rectal NETs rarely cause carcinoid syndrome. Maximum tumour diameter and histological grade are major prognostic factors for rectal NETs: tumours ≤10 mm rarely metastasise, whereas tumours >20 mm have a substantially higher risk of nodal or distant metastasis (6, 19, 20). Radical oncological resection is generally recommended for rectal NETs >20 mm or with muscularis propria invasion or regional lymph-node metastasis, whereas management of 10–20 mm lesions and tumours with G2 grade or lymphovascular invasion should be individualised after local staging and multidisciplinary review (19). In the present patient, the primary tumour measured 25 mm in greatest dimension, invaded the subserosa, showed lymph node metastasis, and was histologically graded as G2; this combination was consistent with a high metastatic risk and the subsequent development of hepatic metastases within four months after surgery.

The discordance between the endoscopic biopsy (G1, Ki-67 2%) and the resection specimen (G2, Ki-67 approximately 20%) may reflect Ki-67 intratumoural heterogeneity and undersampling of small biopsy specimens, a recognised phenomenon in GEP-NETs; in G2 tumours, virtual biopsy studies have shown that approximately 59.9% of samples may be undergraded (21). Hot-spot Ki-67 counting on surgical specimens more accurately reflects tumour biological potential; the WHO 2022 classification and ENETS 2023 guidance recommend that grading be based on the area with the highest proliferative activity in adequately sampled tumour tissue (5, 19). Notably, the marked discordance at initial diagnosis between an indolent G1 biopsy phenotype and highly aggressive MRI staging (mrT3b, MRF+, EMVI+) was an early and clinically meaningful warning sign of marked intratumoural heterogeneity and biopsy sampling error; this finding may also reflect broader tumour heterogeneity relevant to the differing MMR protein-expression patterns observed in the sequential liver biopsies. This case illustrates that imaging-pathology discordance should immediately raise clinical suspicion of biopsy sampling error, particularly in tumours with aggressive imaging features. For tumours with adverse radiological features, clinical decision-making should not rely solely on biopsy-based grading.

### 3.2 MMR deficiency in GEP-NETs: prevalence and clinical significance

MMR deficiency is uncommon overall in GEP-NETs. Studies employing combined IHC and MSI testing have shown that dMMR/MSI-H is very rare in well-differentiated GEP-NETs; in published series of pancreatic and small intestinal NETs, most cases are MMR-proficient/MSS. By contrast, dMMR/MSI-H is more common in GEP-NECs, particularly colorectal NECs, with reported frequencies of approximately 12%-30% (22–24). In reported gastrointestinal dMMR/MSI-H NECs, MLH1/PMS2 loss and MLH1 promoter hypermethylation are common, suggesting that most represent sporadic dMMR; however, mechanistic data in well-differentiated NETs remain limited, and rare Lynch syndrome-associated cases have also been reported (24, 25). Given the rarity of this molecular subtype, prospective ICI data specifically in dMMR/MSI-H NETs remain lacking, and the available prospective evidence is derived mainly from biomarker-unselected NET cohorts, such as KEYNOTE-158 (13).

Current clinical guidelines recommend pembrolizumab for metastatic NETs with confirmed MSI-H/dMMR or TMB-high (>=10 mut/Mb); given the lack of NET-specific prospective data, this recommendation is largely extrapolated from the evidence supporting pan-tumour ICI approvals. The molecular discordance shown in the present case underscores the need for cautious, case-by-case multidisciplinary evaluation.

### 3.3 Progressive MMR protein loss in the context of persistent IHC-NGS discordance: mechanisms and implications

The central observation of this case is that MMR protein loss progressed from partial loss at the first liver biopsy (January 16, 2026; MLH1-, MSH2+, MSH6-, PMS2-) to apparent complete loss of all four proteins at the second biopsy (March 17, 2026; MLH1-, MSH2-, MSH6-, PMS2-). The two biopsies were separated by approximately 60 days, during which the patient received two cycles of carboplatin/etoposide plus surufatinib, while NGS showed persistent MSS and TMB <1 mut/Mb throughout. Biologically, isolated retention of MSH2 with concurrent MSH6 loss at the first biopsy is noteworthy. MSH2 and MSH6 form the MutSα heterodimeric complex; isolated MSH6 protein loss with retained MSH2 is compatible with an MSH6-specific abnormality and has been described in endometrial cancer as a source of IHC-MSI discordance (26). The subsequent complete loss of MSH2 at the second biopsy, without any somatic MMR gene mutation detected by NGS, therefore suggests a non-classical, possibly epigenetic mechanism. Four mechanistic explanations should be considered.

#### 3.3.1 Epigenetic silencing without microsatellite instability

MMR protein loss may arise through epigenetic silencing in the absence of coding mutations. Because IHC and molecular MSI assays assess different consequences of MMR dysfunction, discordant results may occasionally occur (27). MLH1 promoter hypermethylation is a well-established mechanism of sporadic dMMR (28) and has been associated with MLH1 protein loss in gastrointestinal NEC/MANEC (12). When MMR protein loss is focal or subclonal, IHC and bulk molecular testing may yield discordant classifications, although the precise basis is assay- and sample-dependent (29). Platinum exposure can also remodel DNA methylation patterns. In ovarian cancer, platinum-based chemotherapy has been associated with treatment-related methylation changes in blood and recurrent tumour samples (30), while changes involving the hMLH1 promoter and hypermethylation of the hMSH2 upstream regulatory region have been linked to platinum resistance (16, 31). A recent review likewise identified DNA methylation alterations in DNA-repair genes as potential contributors to platinum response and resistance (32). These observations provide a plausible non-mutational framework for MMR protein loss in the present case; however, MLH1 and MSH2 promoter or regulatory-region methylation was not assessed, and an epigenetic mechanism therefore cannot be confirmed.

#### 3.3.2 Clonal heterogeneity and sampling bias

Intratumoural heterogeneity may generate spatially discrete MMR-deficient clones. When such clones are present at low abundance or are unevenly distributed, focal or subclonal MMR protein loss may be detected by IHC, whereas MSI testing or NGS-based algorithms may still classify the tumour as MSS (29). A large concordance study involving 191,767 solid tumour samples found a true discordance rate of 0.31% after pathologist re-evaluation, indicating that a small proportion of tumours show genuine IHC-NGS discordance even after comprehensive molecular profiling (14). A recent colorectal cancer report provides a related gastrointestinal precedent: Khandakar et al. described Lynch syndrome patients who developed MMR-proficient/MSS colorectal adenocarcinomas, including one patient with synchronous colorectal cancers showing different MMR/MSI classifications and divergent responses to pembrolizumab (33). This observation supports the concept that MMR/MSI status may vary across tumour foci and that single-site sampling may fail to capture clinically relevant molecular heterogeneity. The two sequential percutaneous liver biopsies in the present case may therefore have sampled geographically distinct regions of the metastatic burden: one containing a subclone with partial MMR protein loss and another containing a spatially separated subclone with complete loss. Under this interpretation, apparent temporal evolution cannot be definitively distinguished from spatial sampling variation. Because MMR IHC could not be performed on the exhausted primary resection specimen, baseline MMR status remains unavailable and the timing of the observed protein-loss phenotype cannot be established. The very low TMB (<1 mut/Mb) and absence of somatic MMR mutations indicate that the protein loss was not accompanied by a classical hypermutator phenotype, suggesting a non-classical mechanism.

#### 3.3.3 Platinum-associated clonal selection and MMR protein downregulation

The temporal relationship—partial MMR protein loss before chemotherapy followed by apparent complete loss within 60 days after two cycles of carboplatin/etoposide—raises the hypothesis that treatment pressure selected a pre-existing MMR-deficient or low-expression subclone. Platinum compounds form intrastrand DNA adducts that are recognised by MMR complexes, including MutSα (MSH2-MSH6). MMR-proficient cells activate DNA-damage signalling and apoptosis after adduct recognition, whereas MMR-deficient cells may tolerate these lesions and acquire a relative survival advantage during platinum exposure (34, 35). Reviews of platinum resistance similarly identify MMR dysfunction as a mechanism capable of reducing platinum sensitivity (36). Thus, the shift from partial to complete MMR protein loss could reflect enrichment of a pre-existing subclone rather than *de novo* genetic loss. However, therapy-driven clonal selection cannot be distinguished from spatial sampling heterogeneity or functional non-mutational protein downregulation in this case. The absence of somatic MMR mutations and the very low TMB are compatible with a non-classical mechanism but do not establish its cause. Epigenetic silencing, spatial heterogeneity, clonal selection, and functional protein downregulation are not mutually exclusive. Because methylation analysis and baseline MMR IHC of the primary resection specimen were unavailable, the dominant mechanism remains unresolved. This case should therefore be regarded as hypothesis-generating rather than mechanistic proof.

#### 3.3.4 Therapeutic implications of IHC-dMMR/NGS-MSS discordance

Persistent IHC-NGS discordance has direct therapeutic implications. According to current NCCN and CSCO guideline criteria, IHC-defined dMMR may make this patient eligible for pembrolizumab. However, this case reveals a key diagnostic pitfall: the tumour lacks evidence of classical genetic MMR deficiency, yet shows MMR protein loss at the immunophenotypic level. A prospective biomarker analysis of KEYNOTE-158 showed that TMB-H was associated with a higher response rate to pembrolizumab (37). Although dMMR/MSI-H tumours commonly have increased mutation and neoantigen burdens (27), the predictive significance of isolated MMR protein loss in an MSS tumour with very low TMB remains uncertain. Accordingly, the expected benefit of pembrolizumab based solely on IHC-defined dMMR in this setting is uncertain and should be weighed against the potential risk of immune-related adverse events. Emerging evidence further suggests that, in cases discordant between IHC and MSI/NGS, NGS results may predict immunotherapy efficacy better than IHC results in endometrial cancer cohorts, raising the possibility that the same principle may apply to other tumour types (38). Whether patients with IHC-dMMR/NGS-MSS NETs benefit from ICI therapy remains unknown. Prospective biomarker-driven basket trials addressing this subgroup are urgently needed.

### 3.4 Limitations and alternative interpretations

Several important limitations should be acknowledged. First, MMR protein IHC was not performed on the primary surgical resection specimen (C202513774) because the archived FFPE tissue blocks were entirely exhausted during the original diagnostic workflow. Consequently, a baseline MMR assessment is unavailable, and apparent progressive MMR loss remains a clinical inference rather than a verified temporal sequence. Second, the two sequential percutaneous liver biopsies represent spatially limited samples from a heterogeneous metastatic burden, and geographic sampling of distinct MMR-heterogeneous subclones cannot be excluded. Third, both biopsies were processed according to our institution’s standard formalin-fixation protocol. Internal positive controls (stromal fibroblasts, bile duct epithelium, and infiltrating lymphocytes) showed strong and uniform staining in both biopsies; however, in the absence of repeat staining, external review, or whole-slide digital quantification, residual focal technical variability cannot be completely excluded. Fourth, supplementary MMR/MSI molecular testing was not performed, including PCR-based MSI fragment analysis (Bethesda/NCI panel or mononucleotide marker panel) and MLH1/MSH2 promoter methylation analysis; therefore, sustained epigenetic silencing, assay-level discordance, and transient or reversible protein downregulation cannot be distinguished. Fifth, Ki-67 immunostaining was not performed on the second liver biopsy because all available tissue cores were exhausted by the MMR IHC panel and routine H&E sections, leaving no residual material; therefore, an increased proliferation index or transformation to NET G3 cannot be formally assessed at that time point, although the overall morphological pattern remained consistent with a well-differentiated tumour. Sixth, SSTR functional imaging (Ga-68 DOTATATE PET-CT) was not performed, precluding formal assessment of PRRT eligibility.

## 4 Conclusions

To the best of our knowledge, we report the first documented case of metastatic rectal NET Grade 2 showing apparent progressive complete MMR protein loss in the context of sequential platinum-based chemotherapy, with persistent IHC-NGS discordance. This case highlights three clinically important observations: (1) pathological upgrading between endoscopic biopsy and surgical resection is not uncommon in high-risk rectal NETs, and marked imaging-pathology discordance at presentation should raise suspicion of biopsy sampling error; (2) platinum-based chemotherapy may be associated with apparent progressive MMR protein loss, with potential mechanisms including epigenetic remodelling of MMR genes; similar mechanisms have been documented in platinum-resistant ovarian cancer, such as hypermethylation of the hMSH2 upstream regulatory region (16), although spatial sampling heterogeneity cannot be excluded; and (3) persistent IHC-dMMR/NGS-MSS in tumours without evidence of classical genetic MMR deficiency creates a real clinical challenge for immunotherapy decision-making.

Given the single-patient nature of this report, these observations should not be interpreted as evidence supporting routine repeat biomarker testing in all metastatic NETs. These findings suggest that, in metastatic NETs treated with platinum-based therapy, serial molecular or IHC-based MMR assessment may be considered in carefully selected cases when the results would directly affect immunotherapy decisions; however, routine serial invasive biopsy cannot be recommended on the basis of a single case observation. In the absence of NGS-confirmed MSI-H, whether IHC-defined dMMR should confer eligibility for ICI therapy, particularly in tumours without detected somatic MMR-gene alterations, remains unresolved and warrants dedicated prospective research.

## Data Availability

The original contributions presented in the study are included in the article/supplementary material. Further inquiries can be directed to the corresponding author.
